# Application of metagenomic next-generation sequencing in optimizing the diagnosis of ascitic infection in patients with liver cirrhosis

**DOI:** 10.1186/s12879-024-09396-9

**Published:** 2024-05-20

**Authors:** Pei Shi, Juan Liu, An Liang, Wentao Zhu, Jiwei Fu, Xincheng Wu, Yuchen Peng, Songsong Yuan, Xiaoping Wu

**Affiliations:** Department of Infectious Diseases, The First Affiliated Hospital of Nanchang University, No.17 Yongwaizheng Street, Donghu District, Nanchang, Jiangxi Province China

**Keywords:** Metagenomic next-generation sequencing, Ascitic infection, Liver cirrhosis, Culture, Diagnosis

## Abstract

**Background:**

Metagenomic next-generation sequencing (mNGS) is an emerging technique for the clinical diagnosis of infectious disease that has rarely been used for the diagnosis of ascites infection in patients with cirrhosis. This study compared mNGS detection with conventional culture methods for the on etiological diagnosis of cirrhotic ascites and evaluated the clinical effect of mNGS.

**Methods:**

A total of 109 patients with ascites due to cirrhosis were included in the study. We compared mNGS with conventional culture detection by analyzing the diagnostic results, pathogen species and clinical effects. The influence of mNGS on the diagnosis and management of ascites infection in patients with cirrhosis was also evaluated.

**Results:**

Ascites cases were classified into three types: spontaneous bacterial peritonitis (SBP) (16/109, 14.7%), bacterascites (21/109, 19.3%) and sterile ascites (72/109, 66.1%). In addition, 109 patients were assigned to the ascites mNGS-positive group (80/109, 73.4%) or ascites mNGS-negative group (29/109, 26.6%). The percentage of positive mNGS results was significantly greater than that of traditional methods (73.4% vs. 28.4%, *P* < 0.001). mNGS detected 43 strains of bacteria, 9 strains of fungi and 8 strains of viruses. Fourteen bacterial strains and 3 fungal strains were detected via culture methods. Mycobacteria, viruses, and pneumocystis were detected only by the mNGS method. The mNGS assay produced a greater polymicrobial infection rate than the culture method (55% vs. 16%). Considering the polymorphonuclear neutrophil (PMN) counts, the overall percentage of pathogens detected by the two methods was comparable, with 87.5% (14/16) in the PMN ≥ 250/mm^3^ group and 72.0% (67/93) in the PMN < 250/mm^3^ group (*P* > 0.05). Based on the ascites PMN counts combined with the mNGS assay, 72 patients (66.1%) were diagnosed with ascitic fluid infection (AFI) (including SBP and bacterascites), whereas based on the ascites PMN counts combined with the culture assay, 37 patients (33.9%) were diagnosed with AFI (*P* < 0.05). In 60 (55.0%) patients, the mNGS assay produced positive clinical effects; 40 (85.7%) patients had their treatment regimen adjusted, and 48 patients were improved. The coincidence rate of the mNGS results and clinical findings was 75.0% (60/80).

**Conclusions:**

Compared with conventional culture methods, mNGS can improve the detection rate of ascites pathogens, including bacteria, viruses, and fungi, and has significant advantages in the diagnosis of rare pathogens and pathogens that are difficult to culture; moreover, mNGS may be an effective method for improving the diagnosis of ascites infection in patients with cirrhosis, guiding early antibiotic therapy, and for reducing complications related to abdominal infection. In addition, explaining mNGS results will be challenging, especially for guiding the treatment of infectious diseases.

## Introduction

Liver cirrhosis is a chronic progressive liver disease caused by one or more etiologies and characterized by diffuse fibrosis, pseudolobules, and regenerative nodules in liver tissue [1]. Due to the influence of portal hypertension, hypoproteinemia and bacterial translocation, ascites infection is common in cirrhotic patients [2–4]. Ascites is the term used to denote increased fluid in the peritoneal cavity, a situation that is not normal. Ascites is the most common complication of liver cirrhosis and indicates natural course of progression to decompensated liver cirrhosis. Once ascites occurs, the mortality rate is approximately 15% within one year and 44% ∼ 85% within five years [5, 6].

According to the current clinical guidelines, the types of ascites infection in patients with liver cirrhosis can be divided into spontaneous bacterial peritonitis (SBP) and bacterascites [2]. SBP is a typical type of abdominal infection in liver cirrhosis that occurs in approximately 40% ∼ 70% of cirrhotic patients and plays an important role in the progression of liver cirrhosis, the occurrence of liver-related complications and mortality [7, 8]. SBP was defined by polymorphonuclear neutrophil (PMN) count in ascites ≥ 250/mm^3^ with positive or negative microbial culture results. Bacterascites, defined by ascites PMN count < 250/mm^3^ with positive microbial culture results, may represent the first step in the development of SBP [9]. However, research data on bacterascites are rare, and there is an absence of clinical guidelines for the management of bacterascites.

The current diagnostic criteria for SBP are based on ascites PMN count ≥ 250/mm^3^, which has limited guiding significance for clinical practice [10]. On the one hand, the threshold of PMN count in ascites patients is based on a small sample in a retrospective study, and there is no rigorous research support [11]. On the other hand, cell lysis may occur during the transport of ascites samples to the laboratory, resulting in false-negative results [12]. Due to the long culture time and low percentage of positive ascites culture [13], diagnosing abdominal infection in cirrhotic patients with ascites is still difficult. Empirical use of antibiotics is therefore common and may lead to the emergence of drug-resistant bacteria and the occurrence of secondary infections [2, 14]. Therefore, in clinical practice, more sensitive and faster diagnostic methods are needed to identify abdominal infection early and treat them quickly.

Over the past decade, next-generation sequencing (NGS) technology has continued to develop, moving from scientific research to clinical practice, NGS was initially applied for individualized diagnosis and treatment of tumors and for screening of genetic diseases. In recent years, the field of infectious diseases has gradually expanded to including the rapid identification of pathogens and the tracking of the spread and evolution of pathogens [15]. Among these methods, metagenomic next-generation sequencing (mNGS) technology has the advantages of rapid, unbiased detection [16], extensive pathogen detection [15, 17], and high accuracy [18] and has become an important method for detecting new rare and coinfectious pathogens [19, 20]. However, it is unknown whether mNGS is effective in the etiological diagnosis and management of cirrhotic patients with ascitic fluid infection (AFI).

This study retrospectively included 109 patients with cirrhotic ascites, and conventional bacterial and fungal culture and mNGS were applied to detect pathogens in ascites samples. This study aimed to optimize the diagnosis of cirrhotic ascites through mNGS technology and to provide basic data and new ideas for early etiological diagnosis and early treatment of abdominal infections related to cirrhosis.

## Materials and methods

### Patient enrollment

A total of 109 patients with cirrhotic ascites who underwent abdominal puncture at the Department of Infectious Diseases, the First Hospital of Nanchang University, from December 2020 to January 2023 were retrospectively enrolled. The inclusion criteria were as follows: (1) patients with cirrhosis, (2) patients aged older than 18 years and (3) patients with moderate/abundant ascites (ascites depth > 3 cm) at admission. The exclusion criteria were as follows: (1) pregnant women, (2) patients whose ascites could not be extracted through abdominal puncture, (3) patients with liver tumors and extrahepatic-related tumors, (4) patients with secondary peritonitis, (5) nonportal hypertensive ascites, and (6) HIV patients. Cirrhotic ascites was classified into four types as follows [2]: (1) Culture-positive SBP: ascites PMN ≥ 250/mm^3^ with ascites culture positive; (2) Culture-negative SBP: ascites PMN ≥ 250/mm^3^ with ascites culture negative; (3) Bacterascites: ascites PMN < 250/mm^3^ but ascites culture positive; and (4) Sterile ascites: ascites PMN < 250/mm^3^ and ascites culture negative. The patients were divided into three groups according to above types of ascites: (1) SBP: ascites PMN ≥ 250/mm^3^ with ascites culture positive or negative; (2) bacterascites; and (3) sterile ascites.

### Clinical data and sample collection

General information, clinical data and laboratory indexes of patients were collected from the electronic medical records. Ascites samples were taken within 48 h of admission and examined for routine biochemistry, culture and mNGS.

### Isolation, culture and identification

The ascites samples were processed according to microbial culture procedures [21]. Ascites was collected before antimicrobial agents were administered, if possible. Ascites samples from each patient were collected by aseptic methods, injected into blood culture bottles (Aerobic, Anaerobic and Myco/F Lytic bottles, 10 ml per bottle), and immediately sent to the microbiological testing room of our hospital for detection of aerobic bacteria, anaerobic bacteria, mycobacteria, and fungus. VITEK 2 Compact, an automated microbial identification system, from bioMerieux, Inc., was utilized for identification of the microbial population and detection of drug resistance.

### Metagenomic next-generation sequencing

Ascites samples were collected and transported in accordance with the requirements of BGI (The Beijing Genomics Institute). Strict aseptic operation: The disinfectant was applied to the skin for a while and allowed to dry before operation. At least 5 ml of ascites were collected and stored in sterile drying tubes, avoiding collection through drainage tubes. Sterile tubes were sealed with sealing film and transported on dry ice.

DNA extraction: After thawing, 600 µl samples were mixed with 7.2 µl of lytic acid (RT 410, Tiangen Biotechnology) and incubated at 30 °C for 10 min. To purify the DNA, samples were transferred to lysing matrix tubes after brief centrifugation and mixed well in the FastPrep instrument. Then, samples were centrifuged at 2000 rpm for 20 s and 300 µl supernatants were transferred to 1.5 ml centrifuge tubes. A QIAamp DNA Micro Kit (QIAGEN) was used to extract DNA according to standard procedures.

DNA quantification: (1) experiment preparation: The dsDNA BR Assay Kit (Yisheng Biotechnology, Shanghai) was placed in room temperature before use. Prepared and labeled a quantity of 0.5 ml thin-walled PCR tubes. (2) preparation of working solution: Diluted the appropriate amount of dsDNA BR Reagent to 1× with dsDNA BR Buffer and placed it in a lightproof plastic container. (3) preparation of the standard products to be tested: We taken 190 µl working solution into the standard PCR tubes, and added 10 µl of dsDNA BR Standard 1 and dsDNA BR Standard 2 into the corresponding standard PCR tubes. The mixture was gently vortexed for 2–3 s. (4) preparation of the sample to be tested: We taken 180–199 µl working solution into the sample PCR tubes, added 1–20 µl of the sample to be tested, so that the final volume of each sample in the PCR tubes was 200 µl. The mixture was then gently vortexed for 2–3 s. (5) detection: All PCR tubes to be tested were incubated at room temperature away from light for 2 min. We followed the instructions for Qubit 3.0 fluorometer (Life Technologies) and selected the dsDNA BR assay program to measure the fluorescence signal values.

The following mNGS procedure included library construction, sequencing, and bioinformatics analyses. The metagenomic library was constructed by the QIAseq Ultralow Input Library Kit (Illumina, USA). Library quality was monitored by Agilent 2100 (Agilent Technologies, USA). Qualified double-stranded DNA libraries became single-stranded DNA by denaturation and cyclization. By rolling circle amplification (RCA), single-stranded circular DNA was transformed into DNA nanoballs (DNBs). Qubit 2.0 (Thermo Fisher Scientific, USA) was used to control the quality of DNBs. Qualified DNBs were loaded into the flow cell and sequenced on the BGISEQ-500 sequencer. Up to 20 libraries per batch were pooled and sequenced on Nextseq 550 platform (Illumina, USA) [22]. Bioinformatics analysis software was used for filtering out low-quality [23], low-complexity, short read (< 35 bp in length) and human sequence data [24–26]. The remaining high-quality sequencing data were compared with the microbial genome database [screened from NCBI (ftp://ftp.ncbi.nlm.nih.gov/genomes/)] using SnapGene software. The stringent mapped read number (SMRN) that matched each microorganism was counted. The coverage and depth of each microbial comparison were calculated by using Bedtools software [27]. The minimum detection limit of microbial nucleic acid in samples was 100 ∼ 1000 copies/mL, and its specificity and repeatability were greater than 99% and 99%, respectively.

### Statistical analysis

Categorical variables were shown as frequencies (%) and analyzed by the chi‑square test. Continuous variables were expressed as medians (interquartile ranges) and analyzed using the Mann-Whitney U test. SPSS 23.0 software (IBM Corp., Armonk, NY, USA) was used for data analyses. A two-tailed *P* value < 0.05 was considered to indicate statistical significance.

## Results

### Demographic and clinical characteristics

A total of 109 cirrhotic patients with ascites were enrolled, 88 were males (80.7%) and 21 were females (19.3%), with a median age of 54 years. The causes of cirrhosis were as follows: 58 cases of hepatitis B virus (HBV) infection (53.2%), 5 cases of hepatitis C virus (HCV) infection (4.5%), 10 cases of alcoholic liver disease (9.1%), 7 cases of HBV infection plus alcoholic liver disease (6.4%), 6 cases of autoimmune liver disease (5.5%), 3 cases of schistosomiasis cirrhosis (2.7%), and 20 cases of unknown cause (18.3%). Ten patients met the diagnostic criteria for acute-on-chronic liver failure (ACLF) at admission, including 1 with ACLF-1, 8 with ACLF-2 and 1 with ACLF-3. Among the 109 patients, gastrointestinal bleeding occurred in 17 patients (15.5%), 16 patients had with hepatic encephalopathy (14.6%) and 37 patients had hepatorenal syndrome (33.9%). Regarding the analyses of the ascites samples, 16 patients (14.7%) with a PMN ≥ 250/mm^3^ and 93 patients (85.3%) had a PMN < 250/mm^3^ (Table 1).

**Table 1:** Demographic and clinical characteristics of enrolled patients

Characteristic	Value
Male, n (%)	88 (80.7%)
Age (years)	54 (43, 64)
Etiology of cirrhosis, n (%)	
HBV	58 (53.2%)
HCV	5 (4.5%)
Alcohol	10 (9.1%)
HBV plus alcohol	7 (6.4%)
Autoimmune	6 (5.5%)
Schistosomiasis	3 (2.7%)
Unknown	20 (18.3%)
Complications, n (%)	
Gastrointestinal bleeding	17 (15.5%)
Hepatic encephalopathy	16 (14.6%)
Hepatorenal syndrome	37 (33.9%)
ACLF diagnosis at baseline, n (%)	
ACLF-1	14 (12.8%)
ACLF-2	14 (12.8%)
ACLF-3	6 (5.5%)
Laboratory parameters	
WBC (×10^9^/L)	6.6 (3.9, 9.8)
Neutrophil (×10^9^/L)	4.3 (2.6, 7.6)
Haemoglobin (g/L)	97 (80, 114)
Platelets (×10^9^/L)	94 (57.5, 207.5)
CRP (mg/L)	27.6 (16.4, 56.9)
PCT (µg/L)	0.2 (0.1, 0.7)
ALT (U/L)	36.7 (19.9, 88.8)
AST (U/L)	60.0(34.1-122.4)
Albumin (g/dL)	29.2(26.3–32.3)
Total bilirubin (µmol/L)	62.9(22.3-269.9)
PT (s)	16.7 (13.7, 21.7)
PTA (%)	57.0 (37.3, 73.9)
INR	1.4 (1.2, 1.9)
Serum creatinine (µmol/L)	77.8(58.9-115.7)
PMN count in ascites, n (%)	
≥ 250/mm^3^	16 (14.7%)
< 250/mm^3^	93 (85.3%)
Ascites WBC count (×10^6^/L)	60 (30, 260)
Ascites PMN count (×10^6^/L)	13.5 (4, 76.8)
MELD score	17.8 (10.8, 24.9)

HBV: hepatitis B virus; HCV: hepatitis C virus; ACLF: acute-on-chronic liver failure; WBC, white blood cell, CRP, C-reactive protein; PCT, procalcitonin; ALT, alanine aminotransferase; AST, aspartate aminotransferase; PT, prothrombin time; PTA, prothrombin activity; INR, international normalized ratio; PMN, polymorphonuclear neutrophils, MELD, model for end-stage liver disease

Among the 109 enrolled patients, 3 groups were analyzed, including 16 patients with SBP (14.7%), 21 patients with bacterascites (19.3%) and 72 patients with sterile ascites (66.1%). Compared with those in the bacterascites group and sterile ascites group, the ascites leukocyte and neutrophil counts in the SBP group were greater (*P* < 0.001), while the leukocyte and neutrophil count and the C-reactive protein (CRP) and procalcitonin (PCT) levels in the blood were not significantly greater (Table 2).

**Table 2:** Baseline characteristics of enrolled patients according to ascites type

	SBP (*n* = 16)	Bacterascites (*n* = 21)	Sterile ascites (*n* = 72)	*P* value
Male, n (%)	14 (87.5%)	16 (76.2%)	58 (80.6%)	0.67
Age (years)	58 (42.8, 69.3)	57 (49, 62.5)	52 (43, 64)	0.43
Etiology of cirrhosis, n (%)				
HBV	9 (53.2%)	13	36	0.61
HCV	0 (4.5%)	2	3	0.79
Alcohol	2 (9.1%)	1	7	0.67
HBV plus alcohol	1 (6.4%)	0	6	0.20
Autoimmune	1 (5.5%)	2	3	0.54
Schistosomiasis	1 (2.7%)	0	2	0.67
Unknown	2 (18.3%)	3	15	0.63
Complications, n (%)				
Gastrointestinal bleeding	2	6	9	0.23
Hepatic encephalopathy	1	5	10	0.31
Hepatorenal syndrome	8	7	22	0.33
Laboratory parameters				
WBC (×10^9^/L)	7.8 (5.4, 10.0)	7.0 (3.3, 9.2)	6.2 (3.9, 10.3)	0.43
Neutrophil (×10^9^/L)	5.8 (3.4, 7.6)	4.6 (2.0, 7.1)	3.8 (2.5, 7.9)	0.52
Haemoglobin (g/L)	92 (76.8, 113.8)	88 (70.5, 103.5)	93 (82.3, 110.5)	0.25
Platelets (×109/L)	126 (81, 278.3)	97 (69, 108.5)	97.5 (61.5, 217)	0.284
CRP (mg/L)	38.4 (24.9, 92.0)	33.3 (17.1, 64.0)	27.1 (15.0, 49.5)	0.27
PCT (µg/L)	0.2 (0.1, 1.6)	0.2 (0.1, 1.3)	0.2 (0.1, 0.5)	0.76
ALT (U/L)	23.5 (15.0, 106.4)	31.6 (18.9, 72.7)	38 (22.3, 93.2)	0.52
AST(U/L)	32.0 (27.5, 119.9)	64.4 (31.3, 188.7)	60.1 (45.5, 121.3)	0.19
Albumin (g/dL)	30.0 (26.0, 31.8)	27.9 (22.7, 29.8)	29.3 (27.2, 33.0)	0.12
Total bilirubin (µmol/L)	35.7 (10.2, 282.0)	110.6 (40.8, 280.3)	60.5 (20.7, 268.8)	0.22
PT (s)	15.1 (14.0, 21.4)	18.8 (14.5, 21.9)	16.9 (13.4, 21.6)	0.37
PTA	66.7 (44, 73.2)	43.6 (36.4, 65.6)	59.3 (37.7, 76.1)	0.23
INR	1.3 (1.2, 1.9)	1.7 (1.3, 2.0)	1.4 (1.2, 1.9)	0.34
Serum creatinine (µmol/L)	74.3 (59.2, 125.2)	69.1 (56.4, 107.7)	78.1 (59.9, 116)	0.19
Ascites WBC count (×10^6^/L)	1400 (810, 2400)	40 (10, 165) *	50 (30, 97.5) *	< 0.001
Ascites PMN count (×10^6^/L)	900 (414, 2167.8)	7.5 (1.5, 40.25) *	9.5 (4, 30) *	< 0.001
MELD score	17.8 (6.5, 27.4)	20.0 (15.1, 24.8)	15.9 (10.3, 25.1)	0.36

SBP, spontaneous bacterial peritonitis; HBV: hepatitis B virus; HCV: hepatitis C virus; WBC, white blood cell; CRP, C-reactive protein; PCT, procalcitonin; ALT, alanine aminotransferase; AST, aspartate aminotransferase; PT, prothrombin time; PTA, prothrombin activity; INR, international normalized ratio; PMN, polymorphonuclear neutrophils; MELD, model for end-stage liver disease

*P* value from Kruskal-Wallis test for continuous variables or Fisher’s exact test for discrete variables comparing patients with SBP to patients with bacterascites and sterile ascites. *P < 0.05 versus SBP

According to the results of ascites mNGS detection, there were 80 patients in the ascites mNGS-positive group and 29 patients in the ascites mNGS-negative group. There were no statistically significant differences in age, sex, distribution of etiology or occurrence of complications between the two groups. The PCT level in the mNGS-positive group was significantly greater than that in the mNGS-negative group (*P* = 0.01), but there were no significant differences in the leukocyte and neutrophil counts in blood or ascites or in the proportion of SBP at baseline between the two groups (Table 3).

**Table 3:** Baseline characteristics of enrolled patients according to mNGS results

	Ascites mNGS positive (*n* = 80)	Ascites mNGS negative (*n* = 29)	*P* value
Male, n (%)	64 (80%)	24 (82.8%)	0.747
Age (years, mean ± SD)	54.5 (45, 64.75)	50 (42, 63)	0.323
Etiology of cirrhosis, n (%)			
HBV	47	11	0.054
HCV	4	1	0.725
Alcohol	5	5	0.167
HBV plus alcohol	3	4	0.148
Autoimmune	4	2	0.708
Schistosomiasis	3	0	0.563
Unknown	14	6	0.704
Complications, n (%)			
Gastrointestinal bleeding	15	2	0.227
Hepatic encephalopathy	13	3	0.643
Hepatorenal syndrome	29	8	0.399
Bacterial infection	31	7	0.157
ACLF diagnosis at baseline, n (%)			
ACLF-1	10	4	1
ACLF-2	11	3	0.884
ACLF-3	6	0	0.297
Laboratory parameters			
WBC (×10^9^/L)	6.4 (3.6, 10.6)	7.1 (3.9, 9.1)	0.85
Neutrophil (×10^9^/L)	4.5 (2.7, 9.0)	3.9 (2.5, 6.4)	0.44
Haemoglobin (g/L)	93.5 (80.0, 112.0)	108 (86.5, 120.5)	0.09
Platelets (×10^9^/L)	90.0 (56.3, 190.8)	130 (64, 264)	0.13
CRP (mg/L)	27.4 (16.9, 64.0)	28.6 (12.7, 46.1)	0.54
PCT (µg/L)	0.2 (0.1, 0.8)	0.1 (0.1, 0.2)	0.01
ALT (U/L)	38 (18.6, 82.7)	32.8 (23.8, 100.8)	0.66
AST(U/L)	63.9 (32.6, 141.6)	54.1 (39.7, 99.2)	0.31
Albumin (g/dL)	29.1 (25.9, 31.8)	29.9 (26.6, 33.5)	0.29
Total bilirubin (µmol/L)	83.6 (27.5, 307.7)	43 (16.6, 181.1)	0.051
PT (s)	17.7 (14.0, 21.8)	15.4 (12.7, 19.6)	0.054
PTA	51.6 (36.8, 71.9)	69 (46.3, 89.2)	0.02
INR	1.55 (1.2, 2.0)	1.3 (1.1, 1.7)	0.03
Serum creatinine (µmol/L)	77.9 (58.9, 122.8)	77.6 (59.7, 97.9)	0.50
PMN count in ascites, n (%)			
≥ 250/mm^3^	13 (16.3%)	3(10.3%)	0.643
< 250/mm^3^	67 (83.8%)	26(89.7%)	0.643
Ascites WBC count (×10^6^/L)	65 (30, 375)	60 (30, 110)	0.49
Ascites PMN count (×10^6^/L)	13.8 (4.1, 132)	12 (3.8, 31.3)	0.47
MELD score	18.9 (12.8, 25.4)	11.2 (7.8, 21.4)	0.66

mNGS, metagenomic next-generation sequencing; HBV: hepatitis B virus; HCV: hepatitis C virus; ACLF: acute-on-chronic liver failure; WBC, white blood cell, CRP, C-reactive protein; PCT, procalcitonin; ALT, alanine aminotransferase; AST, aspartate aminotransferase; PT, prothrombin time; PTA, prothrombin activity; INR, international normalized ratio; PMN, polymorphonuclear neutrophils, MELD, model for end-stage liver disease

### Comparison of pathogen characteristics between ascites culture and mNGS detection

**Fig. 1:**
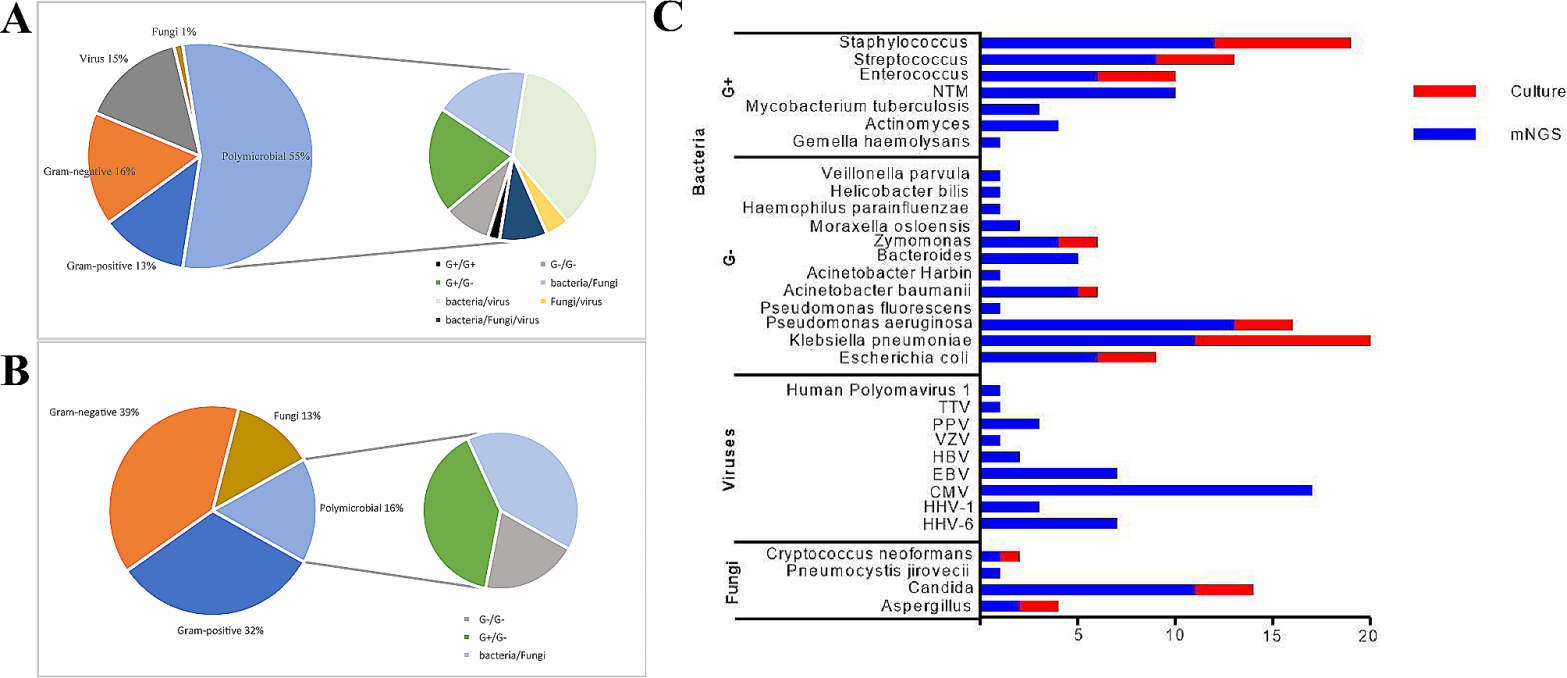
Comparison of pathogens spectrum between ascites culture and mNGS methods. (**A**). mNGS identification for pathogen categories. (**B**). Culture identification for pathogen categories. (**C**). The distribution of detected pathogens of all patients by mNGS and culture methods. mNGS, metagenomic next-generation sequencing

The outcome of ascitic pathogen identification in 109 patients was that the percentage of positive mNGS results was significantly greater than that of traditional methods (73.4% vs. 28.4%, *P* < 0.001). A total of 60 strains of pathogens were detected by mNGS, and 43 strains of bacteria were detected, including 65.1% (28/43) of which were Gram-positive bacteria, 34.9% (15/43) were Gram-negative bacteria, 9 were fungal strains and 8 were viral strains. The top three bacteria according to the detection rate were *Pseudomonas aeruginosa* (13%), *Staphylococcus* (12%) [including *staphylococcus haemolyticus*, *Staphylococcus aureus*, *Staphylococcus epidermidis* and *staphylococcus hominis*] and *Klebsiella pneumoniae* (11%), as shown in Fig. 1A, C. A total of 17 pathogens were detected via the culture method, including 14 strains of bacteria and 3 strains of fungi. The top three bacteria according to the detection rate were *Klebsiella pneumoniae* (8.3%), *Enterococcus faecalis* (3.7%) and *Staphylococcus aureus* (3.7%), as shown in Fig. 1B, C. Mycobacteria [including *Mycobacterium tuberculosis* and nontuberculous mycobacteria (NTM)], viruses (including HHV, EBV, and CMV), and *Pneumocystis* were detected only by the mNGS method (Fig. 1C).

**Fig. 2:**
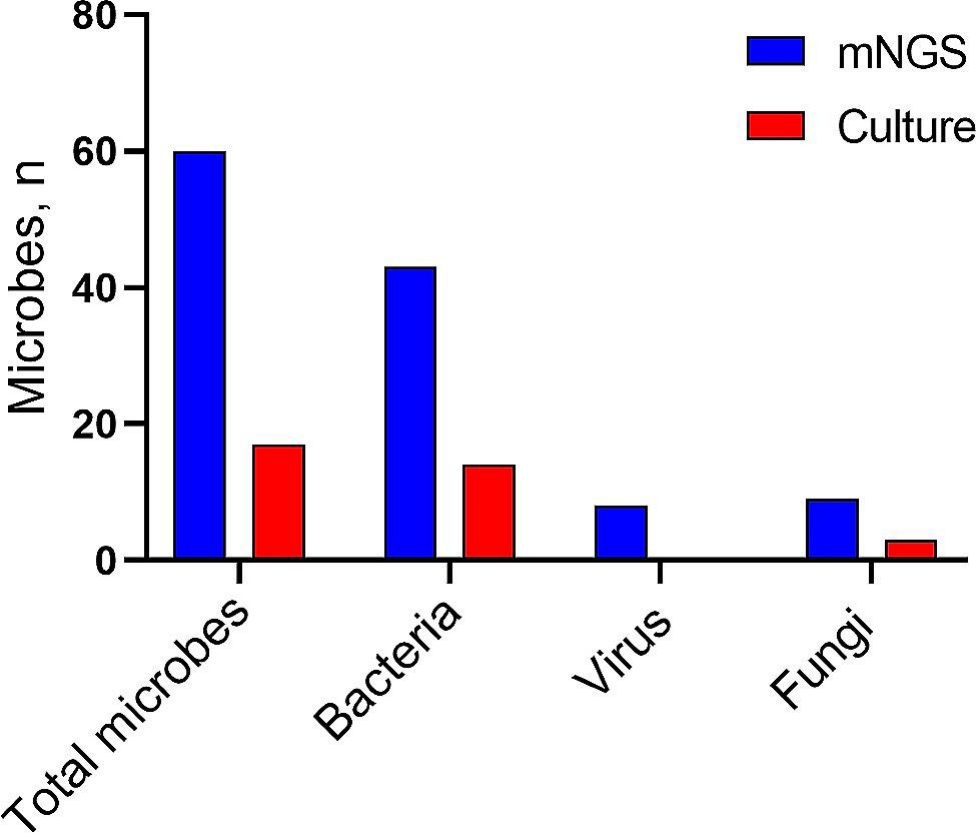
Type of microbes detected by mNGS compared with culture method. mNGS, metagenomic nextgeneration sequencing

The mNGS results showed that the proportion of pathogenic coinfections increased to 55% compared with that of the culture method (16% for culture) (Fig. 1A, B). In 50 of the 109 patients, the culture method failed to identify any microbes, while mNGS detected one (*n* = 19), two (*n* = 19), or three or more (*n* = 12) microbes in each sample. In 29 patients in whom mNGS was negative, only 1 case of monobacterial infection was detected by culture method. mNGS detected more bacterial (43 vs. 174), fungal (9 vs. 3), and viral (8 vs. 0) strains than the culture method (Table 4; Fig. 2). In summary, mNGS detection was more effective than traditional methods in terms of pathogen number and strain type.

**Table 4:** Comparison of microbes detected by mNGS and culture methods for each specimen

		Culture (*n* = 17 microbes)			
		Negative	1	2	3+
mNGS (*n* = 62 microbes)	Negative	28	1	0	0
	1	19	13	1	0
	2	19	4	0	0
	3+	12	8	2	2

mNGS, metagenomic next-generation sequencing

### Comparative analysis of ascitic fluid infection (AFI) diagnosis with mNGS and culture methods

**Fig. 3:**
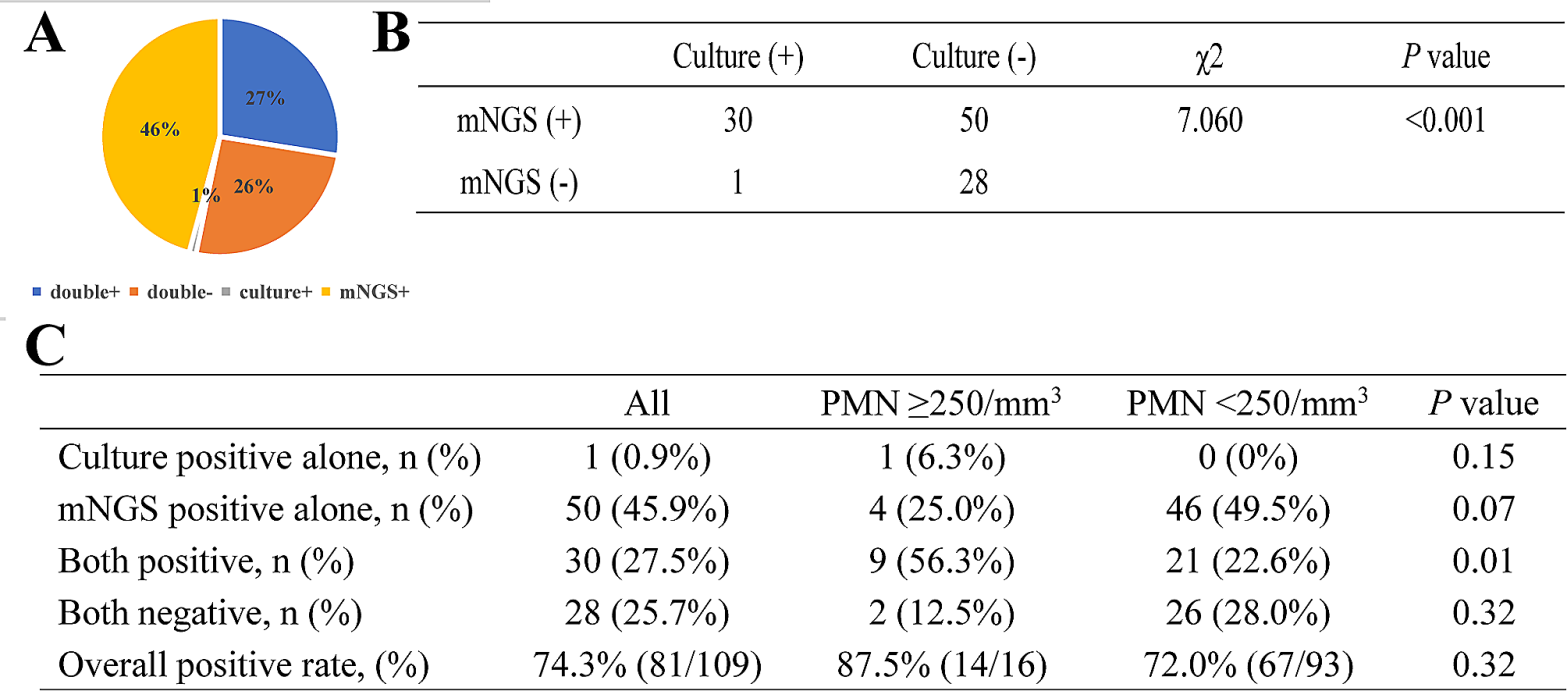
The content of positive results detected by mNGS and culture methods. (**A**). Concordance between mNGS and culture methods for pathogen detection. The pie chart demonstrated the positivity distribution of mNGS and culture for all samples. (**B**). Chi-square test was used to analyze mNGS and culture results of paired samples. (**C**) The positive results of mNGS and culture methods between patients with different PMN count levels. mNGS, metagenomic next-generation sequencing

Among 109 patients with ascites, 30 had positive results, and 28 had negative results (25.7%). mNGS was positive in 50 patients (45.9%), whereas culture detection was positive in only 1 patient (0.9%). A chi-square test of paired mNGS and culture samples was also performed, which revealed that the percentage of positive mNGS results was significantly greater than that of positive culture data (*P* < 0.001) (Fig. 3A, B). In 6 of 30 double-positive patients, the detection results were exactly the same (overlapping all pathogens), which revealed that the percentage of positive mNGS results were completely mismatched (overlap of no pathogen) in 15 of the 30 cases. The other 9 patients were described as “partially matched”, meaning that at least one but not all overlapping pathogens were found in the polymicrobial results.

According to the diagnostic criterion of a PMN ≥ 250/mm^3^ for SBP, 109 samples of ascites were divided into two groups. The overall percentage of positive pathogens detected by the two methods in each group was compared, showing 87.5% (14/16) in the PMN ≥ 250/mm^3^ group and 72.0% (67/93) in the PMN < 250/mm^3^ group (*P* > 0.05) (Fig. 3C).

**Fig. 4:**
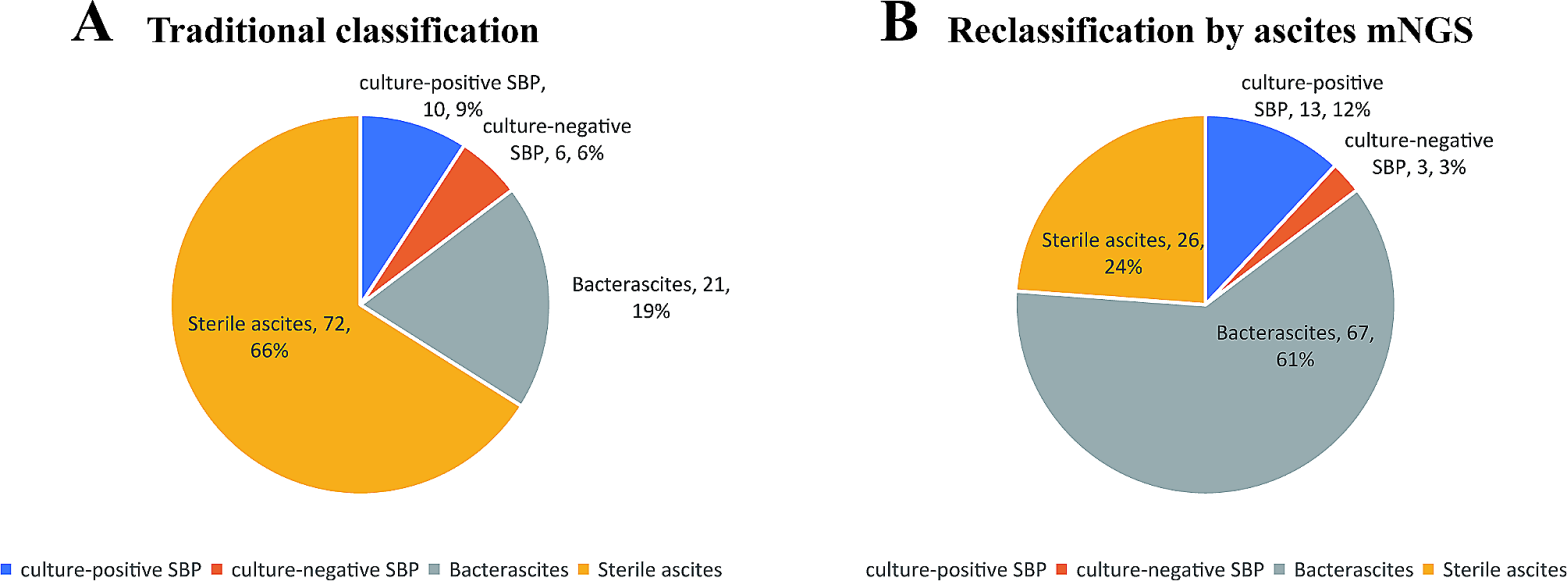
Detection of ascitic fluid infections (AFI) by traditional culture and mNGS methods. (**A**) traditional classification of AFI, (**B**) reclassification of AFI by ascites mNGS. mNGS, metagenomic next-generation sequencing. SBP, spontaneous bacterial peritonitis

We compared the diagnostic accuracy of culture and the mNGS methods for ascitic fluid infection (AFI). The results revealed that 80 patients with positive ascites mNGS results, 30 of whom were positive according to ascites culture and 50 of whom were negative according to ascites culture; 12 patients had an ascites PMN ≥ 250/mm^3^. Among the 29 patients with negative ascites mNGS results, 1 patient had a positive ascites culture result and *Acinetobacter baumannii* infection, 28 patients had a negative culture result, and 2 patients had an ascites PMN ≥ 250/mm^3^. The cirrhotic ascites in this study were classified according to the results of ascites mNGS detection. In the ascites mNGS-positive group, there were 9 patients with culture-positive SBP, 4 patients with culture-negative SBP, 21 patients with bacterascites and 46 patients with sterile ascites. In the mNGS-negative group, 1 patient had a culture-positive SBP, 2 patients had a culture-negative SBP, 0 patient had bacterascites and 26 patients had sterile ascites (Table 5). In summary, 109 samples of cirrhotic ascites were classified according to the ascites culture results: 10 cases of culture-positive SBP, 6 cases of culture-negative SBP, 21 cases of bacterascites, and 72 cases of sterile ascites. On the basis of the mNGS results, the cirrhotic ascites cases were classified as 13 cases of culture-positive SBP, 3 cases of culture-negative SBP, 67 cases of bacterascites, and 26 cases of sterile ascites (Fig. 4).

**Table 5:** The classification of ascitic fluid infection (AFI) according to mNGS results

	Ascites mNGS positive (*n* = 80)	Ascites mNGS negative (*n* = 29)
Culture-positive SBP	9	1
Culture-negative SBP	4	2
Bacterascites	21	0
Sterile ascites	46	26

mNGS, metagenomic next-generation sequencing; SBP, spontaneous bacterial peritonitis

In this study, 72 patients (66.1%) were diagnosed with AFI based on the ascites PMN counts combined with mNGS, and 37 patients (33.9%) were diagnosed with AFI based on the ascites PMN counts combined with culture. There were significant differences in the percentages of patients who were positive for the two diagnostic methods for diagnosing ascites infection (*P* < 0.05) (Table 6; Fig. 4).

**Table 6:** Comparison of classification of ascitic fluid infection (AFI) by mNGS and culture methods

	Ascites mNGS detection (*n* = 109)	Ascites culture detection (*n* = 109)	χ2	*P* value
Ascitic fluid infection (AFI)	72	37	39.225	< 0.001
Culture-positive SBP	13	10		
Culture-negative SBP	3	6		
Bacterascites	67	21		
Sterile ascites	26	72		

mNGS, metagenomic next-generation sequencing; SBP, spontaneous bacterial peritonitis

### Clinical effects of mNGS results on patient management

In 80 patients with positive mNGS results, positive effects of mNGS were observed in 60 patients (for which pathogenic evidence was provided by ascites mNGS), the treatment regimen was adjusted in 40 (85.7%) patients to receive early and precise antibiotic therapy; and the treatment regimen was not adjusted in 20 patients due to the coverage of the original antibiotic regimen. In addition to 4 patients who died and 8 patients who were discharged for serious disease or financial reasons, 48 patients were improved. The coincidence rate of the mNGS results and clinical findings was 75.0% (60/80) (Table 7).

**Table 7:** Clinical effects of mNGS results on diagnosis and management

Clinical effect	Role of mNGS	Treatment changes
Positive effect (*n* = 60; 55.0%)	Guided the clinical therapy (*n* = 40; 36.7%)	Received precise treatment
	Provided definitive diagnosis (*n* = 20; 18.3%)	Empirical treatment continued
Negative effect (*n* = 6; 5.5%)	False-positive result lead to incorrect diagnosis (*n* = 6; 5.5%)	Received incorrect treatment
No effect (*n* = 43; 39.4%)	No additional pathogen detected (*n* = 34; 31.2%)	No changes
	Unclear clinical significant (*n* = 9; 11.0%)	

mNGS, metagenomic next-generation sequencing

However, mNGS failed to detect any additional pathogens in 34 patients. In 9 patients without effects, the significance of positive mNGS results was unknown (for contamination or nonpathogenic bacteria). Notably, mNGS was negative in 6 patients. Among them, 3 patients had bacteroides detected before treatment with antibiotics. However, the clinical efficacy of these antibiotics was ineffective, but the bacterial infections was improved after the empirical antibiotic upgrade. Additionally, there were 3 patients clinically diagnosed with tuberculous peritonitis in which streptococcus was reported, resulting in an inability to change the treatment to an anti-tuberculosis drug in a timely manner (Table 7).

## Discussion

In this study, the mNGS technique was used to detect ascites pathogens to optimize the diagnosis of peritoneal infection in patients with decompensated cirrhosis. The main finding was that the percentage of positive mNGS results was significantly greater than that of culture results (73.4% vs. 28.4%, *P* < 0.001). mNGS improved the detection rate of ascites-related pathogens, including a wider spectrum of pathogens (bacteria, fungi, viruses), which may be related to the differences in the effectiveness of the two detection methods. Culture methods focus on the detection of pathogen function; only some bacteria and fungi are detected, and some pathogens are difficult to culture or cannot be cultured. However, mNGS detection focuses on the type of pathogenic microorganism, and the detection results cover a wide range [28]. In addition, traditional culture technology is easily affected by antibiotic use and contaminating bacteria [29]. Disinfection treatment before sample collection may also affect the results of ascites culture, but has no significant effect on the results of mNGS [30].

Among the positive results of ascites mNGS, the proportion of polymicrobial infection was significantly greater than that of culture-positive results, which was consistent with the findings of a study on mNGS in the diagnosis of hematologic infection [31, 32]. The number of pathogens found on the positive ascites mNGS tests ranged from 1 to 6. The results of the positive ascites culture mostly showed a single pathogen. A possible explanation is that in the culture medium of ascites, the dominant bacteria mainly grew, and the dominant bacteria competed with other bacteria [33].

Studies have shown that the PMN count in ascites fluid is strongly influenced by various factors, especially in cirrhotic ascites patients receiving empirical antibiotic therapy. The diagnostic threshold of a PMN ≥ 250/mm^3^ cannot accurately indicate the presence or absence of spontaneous bacterial peritonitis [34]. Therefore, we divided 109 ascites samples into two groups, using 250/mm^3^ as the PMN cutoff value to calculate the percentage of bacteria-positive samples. The total percentage of patients with a PMN count ≥ 250/mm^3^ group was 87.5% (for the PMN < 250/mm^3^ group, it was 72.0%). The results showed that the percentage of positive pathogens was similar, and the application of mNGS technology significantly improved the percentage of positive pathogens, which was in agreement with a previous study [35].

mNGS revealed 43 species of bacteria from 109 patients, with 65.1% of the species being gram-positive and 34.9% being gram-negative. This finding is quite different from previous reports that ascites infection is dominated by gram-negative bacteria [36]. The possible reason is that the 109 ascites samples were all from patients with decompensated cirrhosis, most of whom were in critical condition, were repeatedly hospitalized, and were often treated with broad-spectrum antibiotics. The empirical drugs for treating abdominal infection are mainly third-generation cephalosporins and carbapenems that target gram-negative bacteria [37]. Given the high cost of mNGS, the sample collection time for mNGS is mostly when patients do not respond to empirical treatment. This approach could lead to an improved detection rate of gram-positive bacteria.

The results showed that the percentage of positive ascites PMN counts combined with ascites mNGS for the diagnosis of ascitic fluid infection (AFI) was significantly greater than that of ascites PMN counts combined with ascites culture (*P* < 0.001). A cohort study evaluating the diagnostic capacity of mNGS for focal infections showed that the percentage of patients with a positive coincidence rate between mNGS and clinical diagnosis was significantly greater than that between mNGS and culture [30]. Early ascites mNGS can early identify additional bacterial ascites cases that have been neglected in the past, and provide corresponding prevention or treatment in a timely manner. However, can the ascites mNGS test replace ascites culture in clinical practice? On the one hand, the positive results of the two methods did not coincide exactly [19]. On the other hand, mNGS cannot provide information on pathogen resistance [38], so it is not clear whether the pathogen is resistant to the antibiotics used. However, ascites culture is accompanied by drug resistance detection. Overall, the combined detection of the two methods can improve the detection rate of ascites pathogens in patients with cirrhosis and guide the application of antibiotics. When the results of two detection methods are consistent, the reliability of the results is greater.

The serum PCT concentration in the ascites mNGS-positive group was significantly greater than that in the ascites mNGS-negative group, and there was no significant difference in the number of secondary bacterial infections between the two groups. The results suggested that the serum PCT had suggestive significance for ascites infection. Several studies have evaluated the diagnostic effect of PCT on SBP, and the research results support PCT as a biomarker of SBP in patients with cirrhosis [39–41], which was further verified by the results of this study. A meta-analysis showed that both CRP and PCT had satisfactory accuracy in the diagnosis of bacterial infection in patients with cirrhosis [42]. A study showed that PCT can be used as an early diagnostic marker of ascites SBP in patients with cirrhosis in the absence of other site infections [43]. Another study compared the PCT and CRP levels in patients who were ascites mNGS positive and ascites mNGS negative, and the results showed that there was no significant difference in the levels of PCT and CRP levels between the two groups [44], which was inconsistent with the results of this study. This could be related to the sample size and disease severity of the included patients, and a larger sample size study is needed to further verify the results.

mNGS has the advantages of high sensitivity, wide pathogen coverage, good stability and time savings, but also has some inevitable shortcomings. First, mNGS has a high cost and relatively long reporting time. Second, the coverage of bacterial and fungal sequencing methods for mNGS is still low, and the drug susceptibility of the pathogens is unknown. Finally, it is difficult for mNGS to distinguish colonization from human or environmental contamination because mNGS detects unbiased and broad-spectrum microbial DNA [45, 46]. Therefore, clinicians should make a comprehensive judgment based on patient history, clinical manifestations and other laboratory indicators.

Inevitably, there are several limitations to our study. On the one hand, in this study, the only sample type analyzed by mNGS was ascites, and blood samples were not tested by mNGS, which may not reflect cirrhosis infection at the same time. In addition, the source of the pathogens detected was not analyzed in detail. Pathogens in ascites may come from the gut or blood. A study simultaneously detected ascites mNGS and plasma mNGS in cirrhotic ascites patients and reported that the virus results obtained via ascites mNGS were consistent with those obtained via plasma mNGS, while the bacterial and fungal results were inconsistent [44, 47]. This finding indicates that the viruses in ascites fluid may have originated from the blood, and the sources of bacteria and fungi may include not only the gut-liver axis but also other pathways. Further research will explore the source of bacteria and fungi in ascites to improve the understanding of the pathogenesis of SBP. On the other hand, at present, studies on the application of the ascites mNGS method in the diagnosis and prognosis of cirrhotic ascites patients are rare. The sample size of this study was small, multicenter clinical trials with large sample sizes are needed to verify the relevant findings, and a long follow-up time is needed to evaluate the value of ascites mNGS detection results in the evaluating the prognosis of cirrhotic ascites patients.

## Conclusion

In conclusion, the mNGS assay showed a broader pathogen spectrum and better sensitivity of pathogen detection for cirrhotic ascites patients, especially for patients with polymicrobial infections. Because of the additional advantages of mNGS, such as rapid results and the decreased impact of antibiotic exposure, the mNGS method can be extended to determine early pathogen diagnosis in cirrhotic ascites patients. However, explaining mNGS results will be challenging for guiding the treatment of infectious diseases.

## Data Availability

The research data involve private patient data so cannot be publicly available on ethical grounds. The data relating to this study are available from the corresponding author based on reasonable request.
